# Emodin Alleviates Liver Fibrosis of Mice by Reducing Infiltration of Gr1^hi^ Monocytes

**DOI:** 10.1155/2018/5738101

**Published:** 2018-03-21

**Authors:** Xiang-An Zhao, Guangmei Chen, Yong Liu, Hongyan Wu, Jin Chen, Yali Xiong, Chen Tian, Bei Jia, Guiyang Wang, Juan Xia, Yuxin Chen, Jian Wang, Xiaomin Yan, Zhaoping Zhang, Rui Huang, Chao Wu

**Affiliations:** ^1^Department of Infectious Diseases, Nanjing Drum Tower Hospital, Clinical College of Traditional Chinese and Western Medicine, Nanjing University of Chinese Medicine, Nanjing, Jiangsu, China; ^2^Department of Infectious Diseases, Affiliated Hospital of Nanjing University of Traditional Chinese Medicine, Nanjing, Jiangsu, China; ^3^Department of Laboratory Medicine, Nanjing Drum Tower Hospital, The Affiliated Hospital of Nanjing University Medical School, Nanjing, Jiangsu, China; ^4^Department of Pathology, Nanjing Drum Tower Hospital, The Affiliated Hospital of Nanjing University Medical School, Nanjing, Jiangsu, China; ^5^Department of Infectious Diseases, Nanjing Drum Tower Hospital, The Affiliated Hospital of Nanjing University Medical School, Nanjing, Jiangsu, China; ^6^Department of Infectious Diseases, Nanjing Drum Tower Hospital, Clinical College of Nanjing Medical University, Nanjing, Jiangsu, China

## Abstract

Emodin, as a major active component of* Rheum palmatum* L. and* Polygonum cuspidatum*, has been reported to have antifibrotic effect. However, the mechanism of emodin on antifibrotic effect for liver fibrosis was still obscure. In the present study, we aimed to investigate whether emodin can alleviate carbon tetrachloride- (CCl_4_-) induced liver fibrosis through reducing infiltration of Gr1^hi^ monocytes. Liver fibrosis was induced by intraperitoneal CCl_4_ injection in mice. Mice in the emodin group received emodin treatment by gavage. Pretreatment with emodin significantly protected mice from liver inflammation and fibrosis revealed by the decreased elevation of serum alanine aminotransferase (ALT) and aspartate aminotransferase (AST), as well as reduced hepatic necrosis and fibrosis by analysis of hematoxylin-eosin (HE) staining, Masson staining, *α*-smooth muscle actin (*α*-SMA), and collagen-I immunohistochemistry staining. Further, compared to CCl_4_ group, mice in the emodin group showed significantly less intrahepatic infiltration of Gr1^hi^ monocytes. Moreover, emodin significantly inhibited hepatic expression of interleukin-1*β* (IL-1*β*), tumor necrosis factor-*α* (TNF-*α*), transforming growth factor-*β*1 (TGF-*β*1), granulin (GRN), monocyte chemoattractant protein 1 (MCP-1), and chemokine ligand 7 (CCL7), which was in line with the decreased numbers of intrahepatic Gr1^hi^ monocytes. In conclusion, emodin can alleviate the degree of liver fibrosis by reducing infiltration of Gr1^hi^ monocytes. These results suggest that emodin is a promising candidate to prevent and treat liver fibrosis.

## 1. Introduction

Liver fibrosis, characterized by excessive deposition of extracellular matrix (ECM), is a pathological change after chronic liver injury including viral infection, toxic damage, metabolic disorders, and alcohol abuse [1]. However, there is still a lack of effective drugs for the treatment of liver fibrosis [2]. Understanding the mechanisms of liver fibrosis is important for prevention and control of liver disease progression.

Different cell types in the liver make a network and regulate liver fibrogenesis [3]. During the past decades, it became apparent that hepatic macrophages play central functions in initiating, perpetuating, and restricting liver fibrosis [1, 4–6]. Hepatic macrophages participate in liver fibrosis through various ways. Importantly, hepatic macrophages mediate the transdifferentiation of hepatic stellate cells (HSCs) into collagen-producing myofibroblasts by secreting several profibrotic factors, such as transforming growth factor-*β*1 (TGF-*β*1), platelet-derived growth factor (PDGF), and granulin (GRN) in liver fibrosis [1, 5, 7–9]. Besides, hepatic macrophages can release many proinflammatory cytokines such as tumor necrosis factor-*α* (TNF-*α*), interleukin-1*β* (IL-1*β*), and IL-6 which may induce liver cellular apoptosis and aggravate liver inflammation [8, 10, 11]. Thus, hepatic macrophages are an attractive target for novel therapeutic approaches to liver fibrosis [12].

Hepatic macrophages comprise resident Kupffer cells (KFs) and infiltrating monocyte-derived cells [13]. During the development of liver fibrosis, macrophage pool of the liver can be rapidly expanded by infiltrating phagocytes that mainly originate from circulating monocytes [14–16]. In mice, two major populations of circulating monocytes exist: Gr1^hi^ (Ly-6C^hi^) monocytes and Gr1^lo^ (Ly-6C^lo^) monocytes. In acute and chronic liver injury of mice, Gr1^hi^ monocytes are massively recruited into the liver [8, 11]. As vigorous secretors of proinflammatory and profibrotic factors including TNF-*α* and TGF-*β*1, they drive inflammation and activate HSC thereby triggering a cascade of events leading to liver fibrosis [10, 17].

The accumulation of monocytes is critically due to chemokines monocyte chemoattractant protein 1 (MCP-1) and chemokine ligand 7 (CCL7) [8, 18–20]. When the liver is damaged, liver cells, HSCs, macrophages, and endothelial cells can secrete MCP-1 and CCL7 which combine with the related receptor-chemokine receptor on peripheral blood monocytes surface and recruit Gr1^hi^ monocytes into liver [8, 21, 22].

In the past two thousand years,* Rheum palmatum* L. and* Polygonum cuspidatum* has been used as Traditional Chinese Medicine to treat liver diseases, which has achieved significant curative effects [23]. As the main active ingredient of* Rheum palmatum* L. and* Polygonum cuspidatum*, emodin has been demonstrated to have anti-inflammatory, antitumor, and antifibrotic effects [24, 25]. Further study suggested that emodin has certain antifibrotic effect on liver fibrosis [26, 27]. However, the mechanism of emodin on the antifibrotic effect for liver fibrosis is still obscure. Therefore, in the present study we investigated whether emodin could alleviate the degree of carbon tetrachloride- (CCl_4_-) induced liver fibrosis by inhibiting infiltration of Gr1^hi^ monocytes.

## 2. Materials and Methods

### 2.1. Mice

Male C57BL/6 mice, 7-8 weeks old, weighing 25 ± 2 g, purchased from Beijing Vital River Experimental Animals Technology (Beijing, China), were used in this study. Mice were housed under laminar airflow hoods in a specific pathogen-free room with a 12 h light and 12 h dark schedule and fed autoclaved chow and water at a controlled temperature of 22 ± 2°C, 50–60% relative humidity.

### 2.2. Experimental Protocol

Mice were randomly assigned to the control group, CCl_4_ group, and emodin group. For induction of liver fibrosis, mice in the emodin group and CCl_4_ group were injected intraperitoneally with 0.6 ml/kg dose of CCl_4_ (CCl_4_ : olive oil = 1 : 4, 3 *μ*l/g CCl_4_ oil) twice weekly for 4 weeks. Mice in the control group were injected with equal volume of olive oil as control. Emodin was dissolved in 0.25% sodium carboxymethyl cellulose (CMC-Na) aqueous solution to prepare the appropriate concentration. Emodin was given by gavage at a dose of 20 mg/kg/d in the emodin group. This dose of emodin is the optimal dose proved by the previous study [27]. For the control group and CCl_4_ group, mice were administrated orally with the same volume of CMC-Na aqueous solution.

After 48 h of the last CCl_4_ administration, mice were sacrificed after anesthesia. Blood samples were collected for ALT and AST detection. The liver tissues of mice were collected.

### 2.3. Liver Function Detection

Blood samples were centrifuged at 4000 rpm/min for 15 min to get serum. ALT and AST levels were determined with clinical testing technique through biochemical instrument (Beckman Coulter, Inc., Brea, CA, USA).

### 2.4. Flow Cytometry

Isolation of liver-infiltrating lymphocytes was performed by an automated, mechanical disaggregation system (Medimachine, Becton Dickinson, USA). The liver samples of mice were washed with PBS twice and then cut into small pieces of 3-4 mm^3^. Five pieces were immediately put in a disposable disaggregator Medicon with 50 *μ*m separator mesh (Becton Dickinson, USA) and then 1 ml PBS was added and processed in the Medimachine System for 1 min. Disaggregated cells were removed and pressed through 70 *μ*m cell strainers to obtain single cell suspensions. Cells were used immediately for flow cytometric analysis. Related antibodies were listed as follows: CD45 (557235, BD Pharmingen, USA), CD11b (557397, BD Pharmingen, USA), Gr1/Ly6C (560595, BD Pharmingen, USA), Ly6G (551460, BD Pharmingen, USA), and F4/80 (25-4801, eBioscience, USA). Flow cytometric analysis was performed on a FACS Aria II (BD Bioscience, USA).

### 2.5. Histopathology

Mouse liver tissues were fixed in paraformaldehyde for 48 h, then dehydrated, and finally embedded with paraffin. Paraffin-embedded liver samples were sectioned to 3 *μ*m thin slices, which were performed with hematoxylin-eosin (HE) staining and Masson staining according to standard protocols.

### 2.6. Immunohistochemistry

3 *μ*m paraffin sections of mouse liver samples were dewaxed and hyalinized. Endogenous peroxidase was blocked with 3% H_2_O_2_. Antigen repair was achieved by boiling in ethylenediaminetetraacetic acid- (EDTA-) alkaline solution. Then, sample sections were incubated with various primary antibody: *α*-smooth muscle actin (*α*-SMA, ab5694, Abcam, USA), collagen-I (ab34710, Abcam, USA), F4/80 (ab111101, Abcam, USA), CD45 (ab10558, Abcam, USA), CD11b (ab13357, Abcam, USA), TGF-*β*1 (ab92486, Abcam, USA), GRN (ab191211, Abcam, USA), MCP-1 (ab25124, Abcam, USA), or CCL7 (orb315556, Biorbyt, UK) overnight at 4°C. Then the sections were stained with secondary antibody (Life Technologies, USA) after multiple flushing with PBS buffer. At last, generally diaminobenzidine (DAB) stained, haematoxylin slightly stained, and neutral balata fixed.

### 2.7. Histological and Immunohistochemical Evaluation

Sections with HE staining were examined under light microscopy by an experienced pathologist in a blinded fashion for liver steatosis, necrosis, and leukocyte infiltration.

For Masson staining and immunohistochemical staining of *α*-SMA and collagen-I, five ×100 magnification images of Masson staining and immunohistochemical staining were captured in each section. Positive staining area of Masson staining (blue), *α*-SMA, or collagen-I immunohistochemical staining (brown) per high-power field (HPF) of each image was converted into pixels by Image-ProPlus software (Media Cybernetics, USA). The percentage of positive area was expressed as a fraction of the total number of pixels, averaged across the 5 different regions per section.

Absolute counts of CD45^+^ cells (leucocytes), F4/80^+^ cells (macrophages), and CD11b^+^ cells (monocytes) per HPF of stained liver sections were manually assessed in 5 different fields per section. All quantification was carried out blinded and without prior knowledge of sections or groups.

### 2.8. Real-Time Gene Expression Analysis

Total RNA was extracted from 50 mg frozen liver tissues using TRIzol according to the manufacturer's instructions (Life Technologies, USA) and then reverse-transcribed to get cDNA by using the PrimeScript RT Master Mix kit (Takara, China) according to the relevant experimental manual. Real-time PCR was performed on Step One Plus Real-Time PCR Systems (Life Technologies, USA) using SYBR Premix Ex Taq kit (Takara, China). All primers and PCR product sizes of this study are listed in Table 1. *β*-Actin was used as an internal control [28].

### 2.9. Statistical Analysis

All data are expressed as the mean ± standard error of the mean (SEM). Statistical analysis was performed using one-way analysis of variance (ANOVA) test by SPSS 22.0 software. *P* < 0.05 was considered to be statistically significant.

## 3. Results

### 3.1. Emodin Attenuated CCl_4_-Caused Liver Inflammation

**Figure 1:**
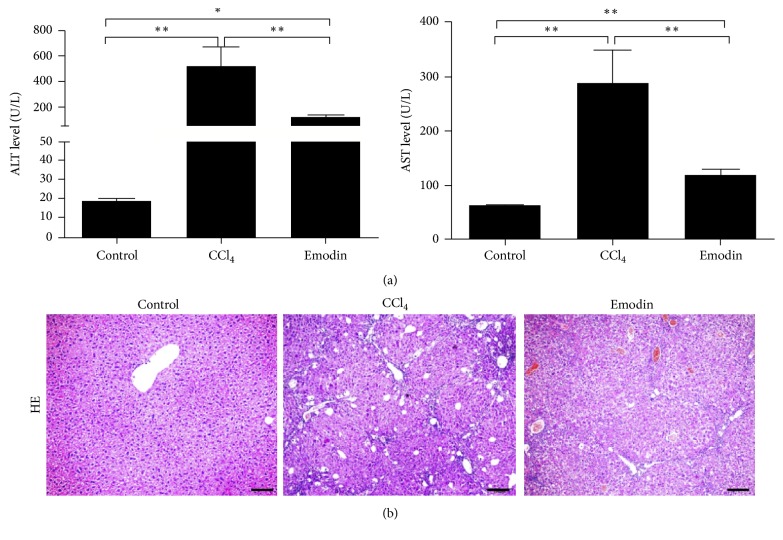
*Emodin attenuated *CCl_4_-*caused liver inflammation*. (a) ALT and AST levels of mice in each group. (b) Hematoxylin-eosin staining of the liver tissues. All data are expressed as the mean ± SEM. ^*∗*^*P* < 0.05; ^*∗∗*^*P* < 0.01. Original magnification: ×100; bar = 50 *μ*m.

The protective effect of emodin on CCl_4_-induced liver injury was identified by liver function and HE staining. As shown in Figure 1(a), the serum ALT and AST increased significantly in mice of CCl_4_ group as compared with the control group. Emodin can significantly reduce the increased liver enzymes. The liver tissues of mice in the CCl_4_ group showed liver steatosis, necrosis, and leukocyte infiltration which were significantly relieved in the emodin group (Figure 1(b)). These results demonstrated that emodin can significantly reduce CCl_4_-induced liver inflammation in vivo.

### 3.2. Emodin Alleviated Liver Fibrosis Induced by CCl_4_

**Figure 2:**
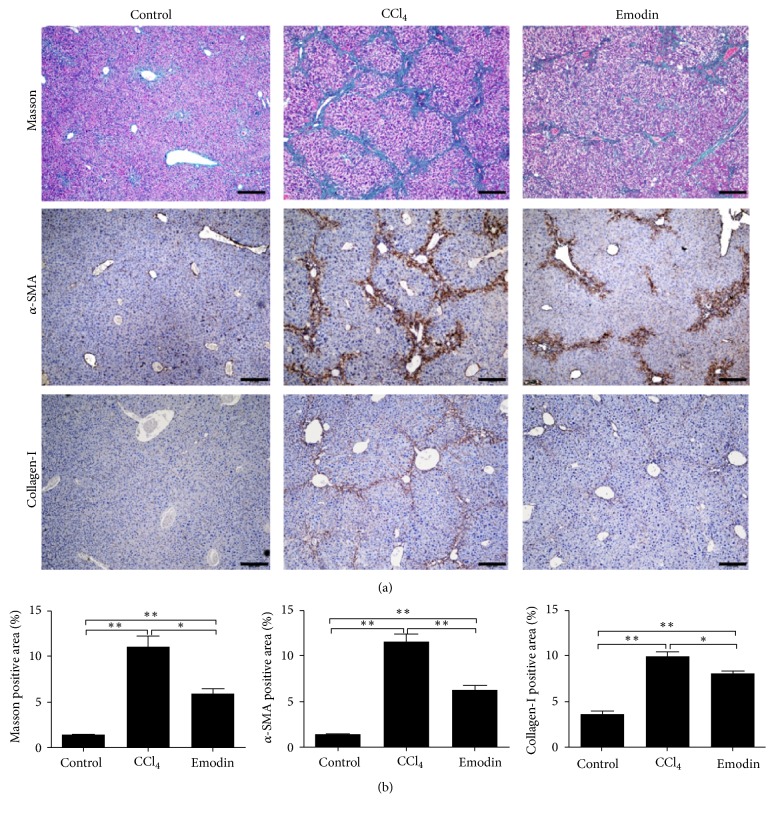
*Emodin reduced *CCl_4_*-caused liver fibrosis in mice*. (a) Masson staining, *α*-SMA staining, and collagen-I staining of the liver tissues. (b) Statistical analyses of collagen deposition. All data are expressed as the mean ± SEM. ^*∗*^*P* < 0.05; ^*∗∗*^*P* < 0.01. Original magnification: ×100; bar = 50 *μ*m.

Masson staining was used to observe the collagen fibers of liver tissues. As shown in Figure 2, fiber staining only appeared on the central venous wall, part of portal area, and interlobular septa in hepatic lobules of control mice. However, compared to the control group, the collagen fibers in CCl_4_ group were significantly higher. The initial formation of interlobular septa and significantly increased portal fiber deposition were observed. The liver tissues of mice in the emodin group also had fiber deposition, mainly distributed at the portal area, fibrous septum, and central vein. But the density of collagen deposition was significantly less than that of CCl_4_ group. Moreover, the liver tissues of mice in the control group had only a small amount of *α*-SMA and collagen-I expression (Figure 2). However, the hepatic expression of *α*-SMA and collagen-I significantly increased in the CCl_4_ group (Figure 2). Emodin treatment can significantly reduce the expression of *α*-SMA and collagen-I induced by CCl_4_ (Figure 2). These results collectively demonstrated that emodin can significantly reduce CCl_4_-induced liver fibrosis in vivo.

### 3.3. Emodin Reduced Gr1^hi^ Monocyte Infiltration

**Figure 3:**
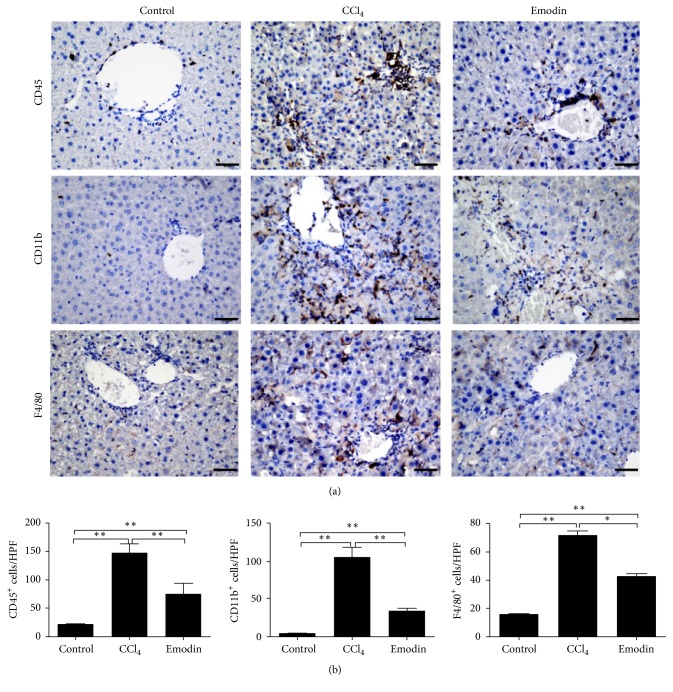
*Emodin reduced infiltrations of leukocytes, monocytes, and macrophages in liver fibrosis*. (a) Immunohistochemistry staining of leukocytes, monocytes, and macrophages in the liver tissues. (b) Statistical analyses of leukocytes, monocytes, and macrophages. All data are expressed as the mean ± SEM. ^*∗*^*P* < 0.05; ^*∗∗*^*P* < 0.01. Original magnification: ×400; bar = 200 *μ*m.

**Figure 4:**
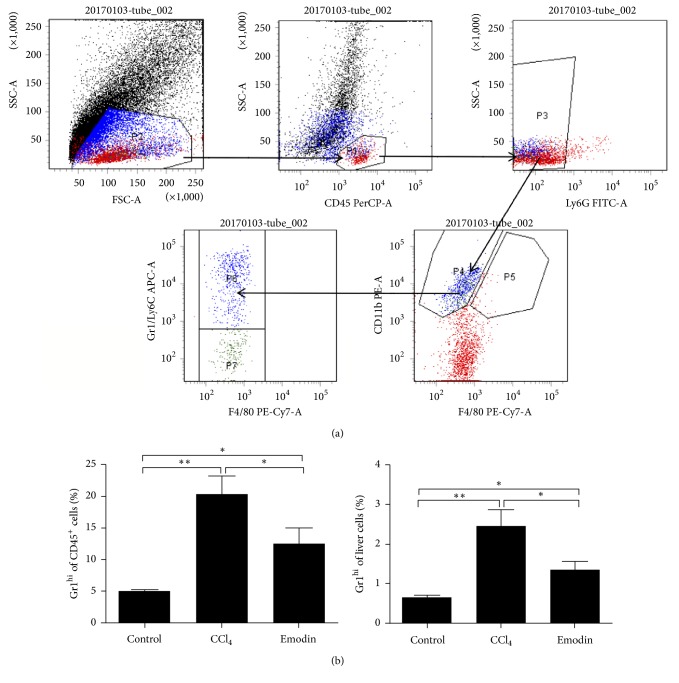
*Emodin reduced infiltrations of *Gr1^hi^* monocytes in liver fibrosis*. (a) Gating strategy of Gr1^hi^ monocytes for flow cytometric analysis. (b) Proportions of Gr1^hi^ monocytes in the liver tissues. All data are expressed as the mean ± SEM. ^*∗*^*P* < 0.05; ^*∗∗*^*P* < 0.01. Original magnification: ×400; bar = 200 *μ*m.

After CCl_4_ challenge for 4 weeks, infiltrations of leukocytes, monocytes, and macrophages increased significantly in the mouse liver tissues of CCl_4_ group. However, compared to CCl_4_ group, mouse liver tissues of emodin group had greatly reduced infiltrations of leukocytes, monocytes, and macrophages (Figures 3(a) and 3(b)). The proportion of Gr1^hi^ monocyte (CD45^+^Ly6G^−^CD11b^+^F4/80^+^Gr1^hi^) subset increased significantly in the liver tissues of CCl_4_ group as compared with the control group. However, the proportion of Gr1^hi^ monocyte subset was significantly lower in the emodin group compared to the CCl_4_ group (Figure 4(b)). These results suggested that emodin can significantly reduce infiltration of monocyte-derived macrophages, especially Gr1^hi^ monocyte in chronic liver injury.

### 3.4. Emodin Inhibited the Expression of Gr1^hi^ Monocyte Associated Proinflammatory and Profibrogenic Cytokines

**Figure 5:**
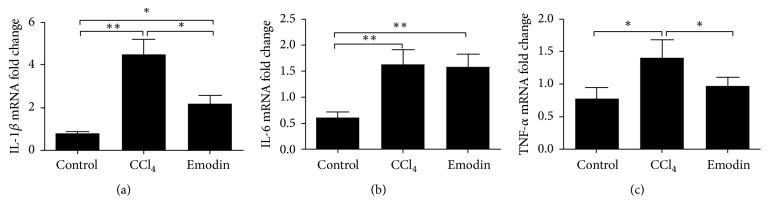
*Emodin inhibited *Gr1^hi^* monocyte associated proinflammatory cytokines*. All data are expressed as the mean ± SEM. ^*∗∗*^*P* < 0.01; ^*∗*^*P* < 0.05.

**Figure 6:**
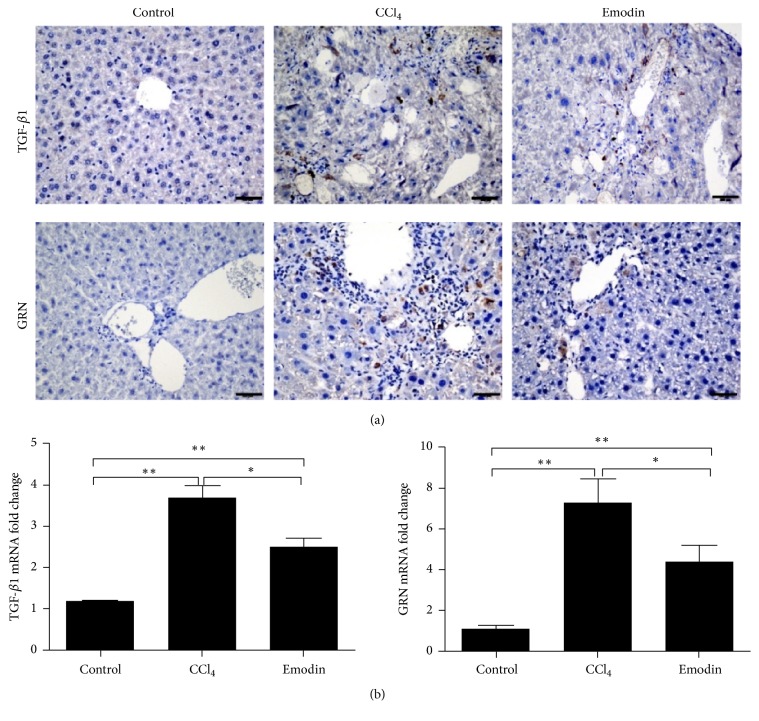
*Emodin inhibited the *Gr1^hi^* monocyte associated profibrogenic cytokines*. (a) Immunohistochemical staining of GRN and TGF-*β*1 in the liver tissues. (b) Hepatic mRNA expression of GRN and TGF-*β*1 in the liver tissues. All data are expressed as the mean ± SEM. ^*∗∗*^*P* < 0.01; ^*∗*^*P* < 0.05. Original magnification: ×400; bar = 200 *μ*m.

Gr1^hi^ monocyte can promote liver fibrosis through releasing many proinflammatory and profibrotic cytokines [1, 5, 7–9]. As shown in Figures 5 and 6, the intrahepatic mRNA expression of IL-1*β*, IL-6, TNF-*α*, TGF-*β*1, and GRN significantly increased after CCl_4_ administration. Mice in the emodin group had significantly lower intrahepatic expression of IL-1*β*, TNF-*α*, GRN, and TGF-*β*1 as compared with CCl_4_ group. As shown in immunohistochemical staining, emodin group had significantly lower expression of TGF-*β*1 and GRN (Figure 6).

### 3.5. Emodin Inhibited the Expression of MCP-1 and CCL7

**Figure 7:**
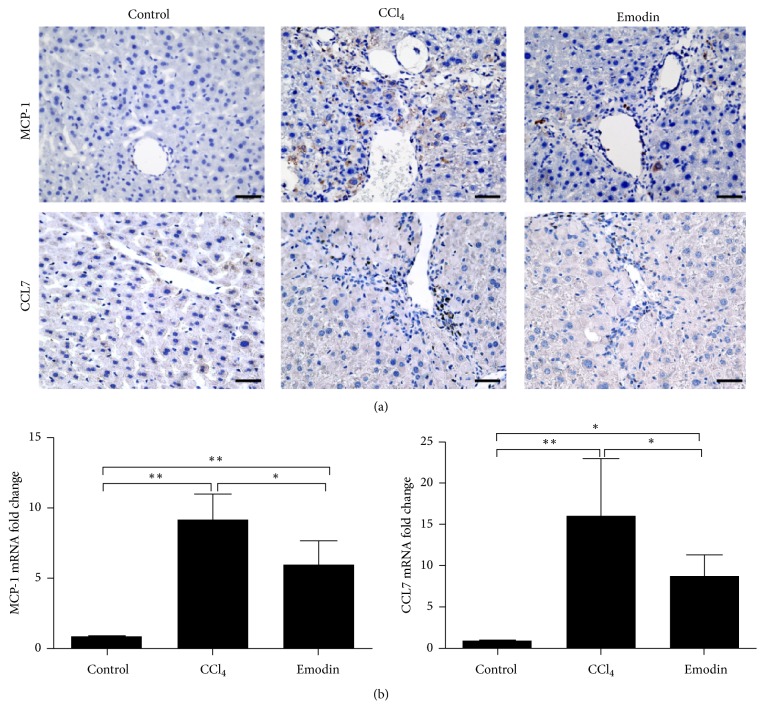
*Emodin inhibited the expression of chemokines MCP-1 and CCL7*. (a) Immunohistochemical staining of MCP-1 and CCL7 in the liver tissues. (b) Hepatic mRNA expression of MCP-1 and CCL7. All data are expressed as the mean ± SEM. ^*∗∗*^*P* < 0.01; ^*∗*^*P* < 0.05. Original magnification: ×400; bar = 200 *μ*m.

MCP-1 and CCL7 are the prime monocyte chemotactic factor [8, 18–20]. Mice in CCl_4_ group have strongly increased hepatic expression of MCP-1 and CCL7, which may be responsible for increasing numbers of intrahepatic Gr1^hi^ monocytes (Figure 7). However, it is obvious that emodin effectively inhibited the hepatic expression of MCP-1 and CCL7 (Figure 7), which may explain why emodin could reduce the infiltration of monocytes.

## 4. Discussion

Hepatic macrophages, especially monocyte-derived macrophages, hold a central position in the pathogenesis of chronic liver injury and have been proposed as potential targets for liver fibrosis [13, 22, 29]. In acute and chronic liver injury, Gr1^hi^ monocytes will firstly be recruited into inflammatory region and release proinflammatory and profibrogenic cytokines which will aggravate liver inflammation and fibrosis of liver [8, 17]. In the present study, we confirmed that, after repeated CCl_4_ injections, Gr1^hi^ monocytes were markedly increased in the liver tissues of mice.

Pharmacological inhibition of Gr1^hi^ hepatic monocytes may be capable of limiting chronic liver injury and fibrosis in vivo [30]. Emodin, as a major active component of* Rheum palmatum *L. and* Polygonum cuspidatum*, has been reported to have anti-inflammatory, antitumor, and antifibrotic effect [24, 25]. Emodin can ameliorate hepatic steatosis through endoplasmic reticulum-stress sterol regulatory element-binding protein 1c pathway in liquid fructose-feeding rats [31]. Liu et al. also reported that emodin could ameliorate liver steatosis and decrease hepatic triglyceride in mice fed with ethanol [32]. In addition, emodin could also significantly inhibit the growth of HSC-T6 cells in vitro [33]. Emodin can also protect the rat liver from CCl_4_-induced liver fibrosis by inhibiting HSCs activation in vivo [27]. In the present study, we also confirmed that emodin could reduce liver inflammation and fibrosis in mouse model induced by CCl_4_. Recent studies show that emodin can inhibit homologous lymphotoxins-induced monocytes migration [34]. On this basis, we used immunohistochemistry staining and flow cytometry to observe the changes of monocytes, especially Gr1^hi^ monocytes. We found that emodin significantly reduced the infiltration of Gr1^hi^ monocytes which is novel mechanism of the antifibrotic effect of emodin.

Gr1^hi^ monocytes can release many proinflammatory and profibrogenic cytokines, including TNF-*α*, IL-1*β*, GRN, and TGF-*β*1. TNF-*α* and IL-1*β* are mainly from monocytes and macrophages in the acute and chronic liver injury [35] and may trigger the production of many other proinflammatory cytokines and induce hepatocyte death through the recruitment of neutrophils [36, 37]. TGF-*β*1 is considered to be the most potent profibrogenic cytokine [38, 39]. TGF-*β*1 can promote liver fibrosis through multiple pathways, for example, activating HSCs, stimulating collagen gene transcription, and suppressing the expression of matrix metalloproteinases [40]. TGF-*β*1 mainly comes from monocyte-derived macrophages [29, 40]. GRNs is a family of protein growth factors that are involved in cell proliferation. Macrophage-secreted granulin may activate resident HSCs into myofibroblasts resulting in a fibrotic microenvironment [7]. In our present study, we found that the hepatic expression of proinflammatory and profibrogenic cytokines IL-1*β*, IL-6, TNF-*α*, TGF-*β*1, and GRN was significantly increased following CCl_4_ injection. However, emodin administration was able to decrease the hepatic expression of IL-1*β*, TNF-*α*, GRN, and TGF-*β*1 in the liver fibrosis model, which also explains why emodin can alleviate liver inflammation and fibrosis.

CCL7 and MCP-1 are the major cytokines for the recruitment of Gr1^hi^ monocytes in the liver [41, 42]. MCP-1 has been well studied in liver fibrosis. In acute and chronic liver injury, MCP-1 can induce liver fibrosis through recruitment of Gr1^hi^ monocytes [28]. In the present study, we also found that the hepatic expression of MCP-1 was significantly higher in the CCl_4_ group as compared with the control group. In Balb/C mice fed on the methionine/choline-deficient diet, a lack of MCP-1 was associated with lower ALT levels and reduced infiltration of inflammatory cells, together with a lower degree of liver fibrosis [43]. Pharmacological inhibition of MCP-1 by mNOX-E36 can also significantly reduce the infiltration of Gr1^hi^ monocytes in the process of acute and chronic liver injury and can reduce the degree of liver fibrosis in vivo [21, 30, 44]. CCL7, previously known as monocyte chemotactic protein-3, belongs to the MCP subfamily of CCLs. CCL7 is expressed at multiple sites of inflammation and is produced by monocytes, fibroblasts, endothelial cells, and mast cells [45–48]. Compared to MCP-1-deficient mice, CCL7-deficient mice showed less infiltration of monocytes in the encephalitis model caused by West Nile virus infection, which indicates that CCL7 has a stronger chemotactic activity than that of MCP-1 [49]. Inhibition of CCL7 could significantly reduce the infiltration of monocytes/macrophages to lung tissues of mice infected with rhinovirus [50]. Our study also found that MCP-1 and CCL7 expression increased significantly during liver fibrosis, suggesting that MCP-1 and CCL7 may play important roles in liver fibrosis. After emodin treatment, the hepatic expression of MCP-1 and CCL7 was significantly reduced which was in line with the reduction of Gr1^hi^ monocytes infiltration. However, the related mechanisms regarding how emodin reduces the secretion of MCP-1 and CCL7 needs further investigation.

In conclusion, we found that emodin has inhibitory effects on liver fibrosis, which may be associated with reducing the Gr1^hi^ monocytes infiltration by the inhibition of MCP-1 and CCL7. These results suggest that emodin may be considered a promising candidate in the prevention and treatment of liver fibrosis. However, the clinical effect of emodin on liver fibrosis deserves further investigation.

## Figures and Tables

**Table 1:** Sequences of primers used for real-time PCR [28].

Gene	Direction	Primer sequence (5′-3′)
TGF-*β*1	Forward	GTGGAAATCAACGGGATCAG
	Reverse	ACTTCCAACCCAGGTCCTTC
GRN	Forward	GCTACAGACTTAAGGAACTC
	Reverse	GAAATGGCAGTTTGATACGG
MCP-1	Forward	ATTGGGATCATCTTGCTGGT
	Reverse	CCTGCTGTTCACAGTTGCC
CCL7	Forward	CGTCCCGTAGACAAAATGGTGAA
	Reverse	GCCGTGAGTGGAGTCATACTGGAACA
IL-1*β*	Forward	GGTCAAAGGTTTGGAAGCAG
	Reverse	TGTGAAATGCCACCTTTTGA
IL-6	Forward	CATTTCCACGATTTCCCAGA
	Reverse	TCCCTCTGTGATCTGGGAAG
TNF-*α*	Forward	AGGGTCTGGGCCATAGAACT
	Reverse	CCACCACGCTCTTCTGTCTAC
*β*-Actin	Forward	GGCTGTATTCCCCTCCATCG
	Reverse	CCAGTTGGTAACAATGCCATGT
